# A Supramolecular Assembly of Hemoproteins Formed in a Star-Shaped Structure via Heme–Heme Pocket Interactions

**DOI:** 10.3390/ijms22031012

**Published:** 2021-01-20

**Authors:** Julian Wong Soon, Koji Oohora, Shota Hirayama, Takashi Hayashi

**Affiliations:** Department of Applied Chemistry, Graduate School of Engineering, Osaka University, 2-1 Yamadaoka, Suita 565-0871, Japan; wongsjuli@chem.eng.osaka-u.ac.jp (J.W.S.); s_hirayama@chem.eng.osaka-u.ac.jp (S.H.)

**Keywords:** cytochrome *b*_562_, hexameric tyrosine-coordinated hemoprotein, supramolecular assembly, heme

## Abstract

Proteins have been used as building blocks to provide various supramolecular structures in efforts to develop nano-biomaterials possessing broad biological functionalities. A series of unique structures have been obtained from the engineering of hemoproteins which contain the iron porphyrin known as heme, as a prosthetic group. This work in developing assembling systems is extended using cytochrome b_562_, a small electron transfer hemoprotein engineered to include an externally-attached heme moiety. The engineered units, which form a one-dimensional assembly via interprotein heme–heme pocket interactions, are conjugated to an apo-form of hexameric tyrosine-coordinated hemoprotein (apoHTHP) to provide a branching unit promoting the assembly of a star-shaped structure. The incorporation of the heme moiety attached to the protein surface of cytochrome b_562_ into apoHTHP can be accelerated by elevating the reaction temperature to generate a new assembly. The formation of a new larger assembly structure was confirmed by size exclusion chromatography. The ratio of the heme-containing units in the assemblies was analyzed by UV-Vis spectroscopy and the population of protein units estimated from SDS PAGE suggests the presence of plausible star-shaped structures, which are supported by hydrodynamic diameter data obtained by dynamic light scattering.

## 1. Introduction

Proteins, known as biofunctional materials, are present in nature and provide essential biological functions which sustain life at the cellular level. In the past two decades, significant attention has turned towards the development of new functionalized nanomaterials via controllable mechanisms which use proteins as building blocks [1,2,3,4]. These artificial assembly systems of proteins can potentially lead to the development of smart biomolecules [5]. The selection of building blocks and techniques to construct such systems has proven to be of great importance in providing controlled structures, new functions and materials with useful properties. These findings contribute to the construction of biomaterials for biomedical applications. Controlling native protein–protein assemblies via electrostatic, hydrogen bonding and hydrophobic interactions is challenging, and such strategies generally have not been successful in producing artificial assembly systems [5]. Therefore, other approaches have been extensively explored in efforts to obtain more ordered and controlled structures using coordination chemistry, host–guest and protein–ligand interactions [6,7,8]. These alternative approaches have been successful in the construction of supramolecular assemblies for studies of protein assembling structures [9,10,11,12].

**Figure 1:**
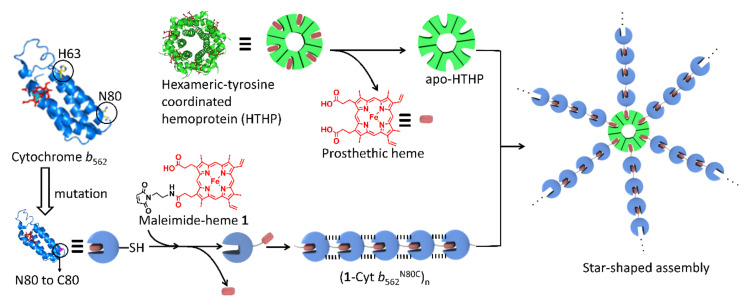
Schematic representation of star-shaped assembly system obtained by the incorporation of the heme moieties from the rigid linear assembly of (**1**-Cyt *b*_562_^N80C^)_n_ into apoHTHP (apo-form of hexameric tyrosine-coordinated hemoprotein).

In developing various artificial protein assemblies, hemoproteins such as myoglobin, horseradish peroxidase, cytochrome *c* and cytochrome *b*_562_ (Cyt *b*_562_) have been investigated by several research groups because of their unique structures, stability, function, reactivity and spectroscopic properties. Our group has focused on the interaction between a given apo-hemoprotein and heme to drive the assembly of the hemoprotein and we have reported on several artificial assembly structures. In our previous investigations, two main strategies have been utilized: (i) self-assembly of an engineered apo-hemoprotein based on an apo-hemoprotein having a covalently attached heme moiety on the protein surface, and (ii) assembly of an apoprotein with a synthetic dimer or trimer of heme moieties [13,14,15,16]. The former strategy was mainly investigated in our first attempt, involving the utilization of a mutant of Cyt *b*_562_, a small electron transfer hemoprotein which contains no cysteine residue in its wild-type form, to include an H63C mutation (Figure 1) yielding Cyt *b*_562_^H63C^ having a cysteine residue at the 63rd position. A maleimide tethered heme **1** (Figure 1) is conjugated to the introduced Cys residue to afford the building block and a supramolecular assembly is formed via successive heme–heme pocket interactions triggered by removal of the endogenous heme to generate the apoprotein. In a more recent study, we have focused on a different engineered Cyt *b*_562_ mutant where a cysteine residue is inserted at the 80th position, Cyt *b*_562_^N80C^ (Figure 1), and its interactions with synthetic heme analogues [17,18]. In contrast to the flexible structure of the assembly based on Cyt *b*_562_^H63C^, the resulting assembly systems generate unique rigid linear and ring shapes, which are dependent on the lengths of the linkers extending between the synthetic heme analogues and protein surface [15]. The short linker generated from ethylene diamine provides a rigid linear assembly of Cyt *b*_562_, (**1**-Cyt *b*_562_^N80C^)_n_, (Figure 1), while the longer linker provides a ring-shaped trimer under low concentration conditions [17,18]. In (**1**-Cyt *b*_562_^N80C^)_n_, the additional electrostatic interactions between the specific residues assist the heme–heme pocket interaction. We have also investigated the assembly of a hexameric tyrosine coordinated hemoprotein (HTHP) with chemical modifications. HTHP is a ring-shaped homohexameric protein (Figure 1). It is considered as an interesting building block for artificial protein assemblies due to its symmetric structure and thermal stability with a denaturation midpoint, *T*_m_, above 130 °C. Chemical modifications of HTHP, via an engineered cysteine residue, enable construction of various assemblies such as a stacked dimer, a two-dimensional sheet, and a micelle-like structure [19,20,21]. In addition to these features, HTHP allows the replacement of heme with artificial cofactors [21,22,23]. Thus, HTHP is one of the useful building blocks for the generation of supramolecular assemblies. 

Although a series of supramolecular hemoprotein assemblies have been reported, structural variations are limited relative to the supramolecular assemblies based on small molecules. Previously, we demonstrated the generation of branched network structures based on a heme trimer and a Cyt *b*_562_^H63C^-based linear assembly, where the additional heme–heme interaction includes a *µ*-oxo dimer of external heme molecules. This system generates massive assemblies with average diameters greater than 1 µm. In contrast to branching in this system using the heme trimer, we expected that a simple star-shaped structure would be obtained by branching with HTHP. In this work, we focus on the conjugation of (**1**-Cyt *b*_562_^N80C^)_n_ and the apo-form of HTHP (apoHTHP) prepared by removal of heme from HTHP toward the new assembly, which forms a star-shaped structure that is promoted by the heme–heme pocket interaction, as shown in Figure 1.

## 2. Results and Discussion

### 2.1. Heme Transfer from Unmodified Cytochrome b_562_ to apoHTHP

**Figure 2:**
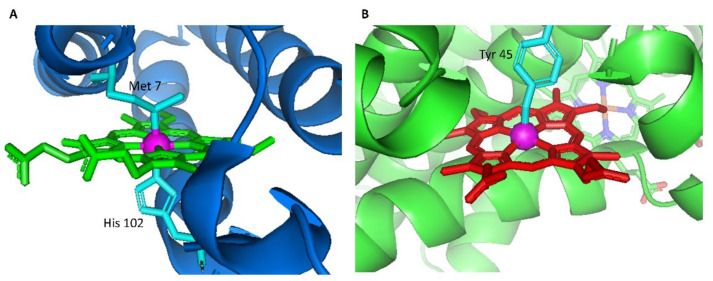
Structural details of heme and its axial ligands in Cyt *b*_562_ (PDB ID: 1QPU, (**A**)) and HTHP (PDB ID: 2OYY, (**B**)).

Prior to formation of the targeted conjugate, heme binding behavior of two building block proteins, Cyt *b*_562_ and HTHP, was qualitatively evaluated. These two proteins are known to have their distinct UV-Vis absorption spectra influenced primarily by their respective axial ligands. The spectra of Cyt *b*_562_ are derived from a low spin hexa-coordinated heme species with His102 and Met7 axial residues (Figure 2A), while the HTHP spectra are generated by a penta-coordinated high spin heme species ligated by a Tyr45 axial residue (Figure 2B). 

**Figure 3:**
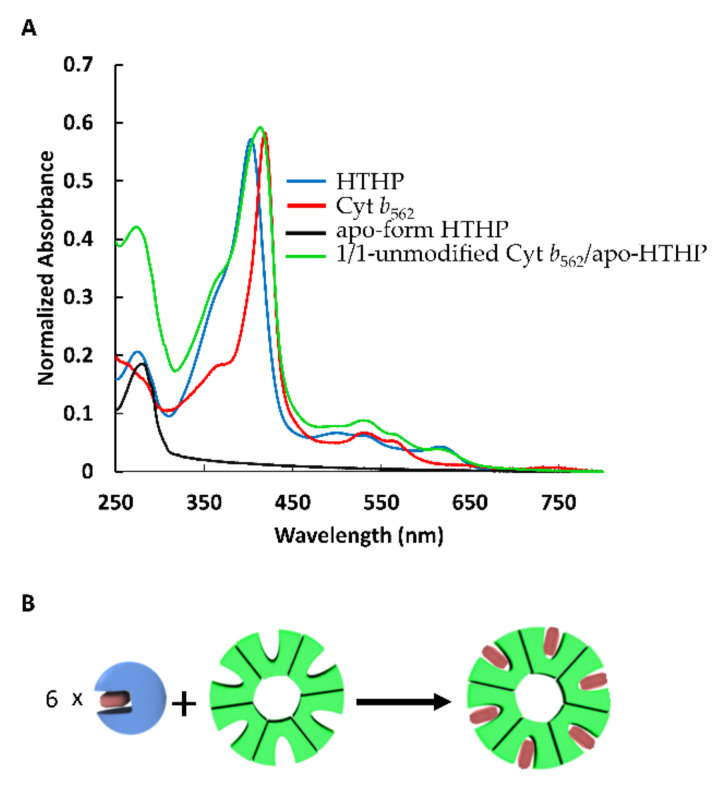
(**A**) The UV-Vis spectra measured at pH 7.0 in 0.1 M potassium phosphate buffer of HTHP (light blue), Cyt *b*_562_ (red), apoHTHP (black), and equimolar mixture of Cyt *b*_562_ and apoHTHP in the same amount as that of the heme binding site (green). (**B**) Schematic representation for the mixing of equivalent unmodified Cyt *b*_562_ and apoHTHP.

The UV-vis spectrum of the ferric state of Cyt *b*_562_ has characteristic absorption peaks at 417 nm, 532 nm and 562 nm [24], while absorption peaks of a ferric state of HTHP are typically observed at 402 nm, 500 nm, 534.5 nm and 623 nm [25,26] (Figure 3A). Thus, ferric UV-Vis spectra can conveniently distinguish the presence of the individual proteins when their apo-forms are mixed with sub-equivalent concentrations of heme molecules. Furthermore, we expected the binding affinity of heme for apoHTHP to be higher than that of apo-Cyt *b*_562_ because *T*_m_ of HTHP is quite high relative to that of Cyt *b*_562_ and *T*_m_ is known to be a good indicator of heme-binding affinity for several hemoproteins [27,28]. The decrease in the binding affinity of native heme for the apo-form of Cyt *b*_562_ was found to be from 19.0 × 10^7^ M at 35 °C to 5.7 × 10^7^ M at 45 °C [27]. Taking this into account, at 45 °C the artificial heme is expected to have a decreased binding affinity for the apo-form of Cyt *b*_562_^N80C^. In contrast, HTHP is thermally stable at well over 45 °C [25] with the *T*_m_ value of around 130 °C. Then, it is likely that a competitive binding affinity of the thermally stable apoHTHP towards the exposed artificial heme on the surface of Cyt *b*_562_^N80C^ at the ends of the linear (**1**-Cyt *b*_562_^N80C^)_n_ is favorable, allowing the binding of the artificial heme to HTHP. Thus, apoHTHP prepared from the wild-type protein indicates absence of absorbance in the visible region in the UV-Vis spectrum (Figure 3A) [21]. Then, unmodified Cyt *b*_562_ containing a prosthetic heme was mixed with apoHTHP under an equimolar condition in the same amount as that of the heme-binding site, as shown in Figure 3B. 

A UV-Vis spectrum of the resulting mixture in Figure 3A indicates a blue shift of the intense Soret band near 400 nm and an increase in absorbance near 630 nm relative to the Cyt *b*_562_ spectrum. According to the aforementioned UV-Vis spectra, the Soret band of HTHP appears at a shorter wavelength than that of Cyt *b*_562_ and the absorbance at 630 nm is typical for HTHP. Thus, these spectral changes clearly indicate the transfer of heme from Cyt *b*_562_ into apoHTHP. Therefore, we expected to observe the formation of the star-shaped assembly upon the addition of apoHTHP into a (**1**-Cyt *b*_562_^N80C^)_n_ solution.

### 2.2. Assembly of Modified Cytochrome b_562_ with apo-form of HTHP

#### 2.2.1. Assembly of (**1**-Cyt *b*_562_^N80C^)_n_ with apoHTHP 

**Figure 4:**
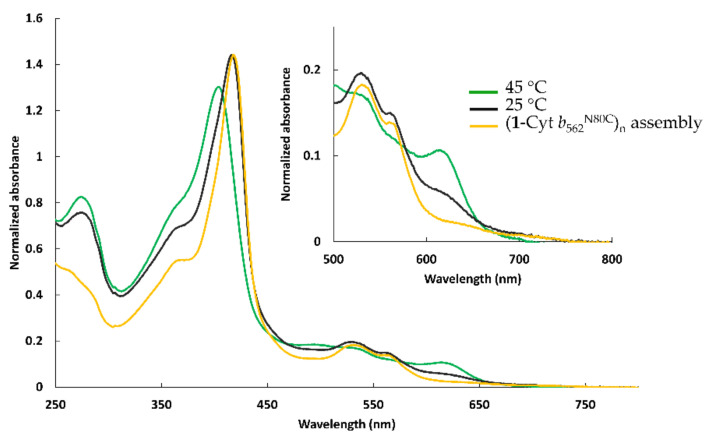
UV-Vis spectra measured at pH 7.0 in 0.1 M potassium phosphate buffer of mixture of (**1**-Cyt *b*_562_^N80C^)_n_ and apoHTHP under the equimolar conditions with respect to the amount of heme-binding site at 25 °C (black) and at 45 °C (green) with UV-Vis spectrum of (**1**-Cyt *b*_562_^N80C^)_n_ (yellow).

Initially, an attempt to mix (**1**-Cyt *b*_562_^N80C^)_n_ and apoHTHP at 25 °C was carried out under equimolar conditions with respect to the amount of heme-binding site in an effort to conjugate the two proteins. However, a UV-Vis spectrum similar to that of (**1**-Cyt *b*_562_^N80C^)_n_ was observed and maintained after 24 h, although a slight blue shift of the Soret band and an increase in absorbance near 630 nm were observed (Figure 4). Thus, we concluded that the release of the heme moiety and/or the binding into apoHTHP generally does not occur at 25 °C. Next, the formation of the assembly was carried out at 45 °C under equimolar conditions with respect to the amount of heme-binding site. Under these elevated temperature conditions, significant spectral changes were observed after 4 h (Figure 4). The characteristic visible absorption peak near 630 nm became prominent. This peak, which is absent in the UV-Vis spectrum of (**1**-Cyt *b*_562_^N80C^)_n_, may be derived from the tyrosine-coordinated heme moiety in the protein matrix of HTHP. The shift of the absorption maximum of the Soret band from 417 nm to 411 nm also indicates that the heme moieties attached to the surface of the Cyt *b*_562_^N80C^ mutant are incorporated into the heme binding sites of HTHP. These findings indicate that increased temperature is required to overcome a kinetic barrier to dissociate the interprotein heme–heme pocket interaction and hydrogen-bonding interactions on the surfaces of the **1**-Cyt *b*_562_^N80C^ units, because incorporation of the heme moiety into the heme pocket of apoHTHP occurs smoothly at the higher temperature. A flexible assembly with a moderately long linker between heme and the protein mutant exhibits a denaturing temperature, *T*_m_, value of ca. 55 °C [15]. The *T*_m_ value of wild-type Cyt *b*_562_ is about. 66.5 °C [24] and we suspect that denaturation of Cyt *b*_562_ does not occur in our system because the reaction temperature is well below both reported temperatures. However, previous studies have indicated that *T*_m_ has an effect on the binding affinity of heme for Cyt *b*_562_ [26]. On the other hand, the thermostability of HTHP may also contribute to the heme–heme pocket interaction resulting in conjugation of the artificial heme to its binding site.

#### 2.2.2. Size Exclusion Chromatography Analysis

**Figure 5:**
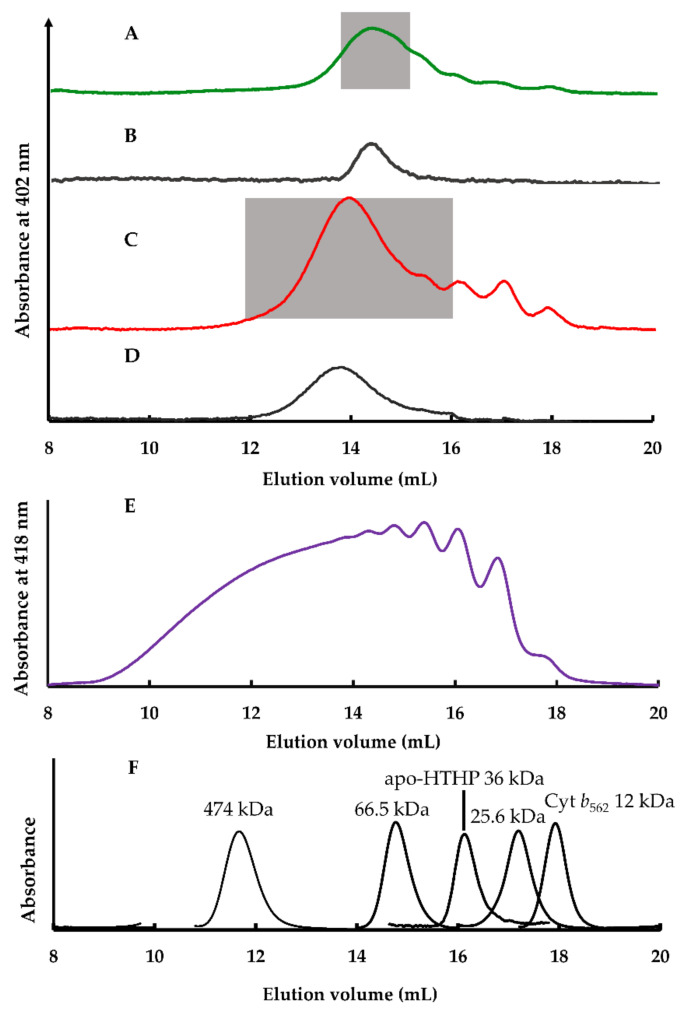
Size exclusion chromatography (SEC) traces of (**A**) a mixture of (**1**-Cyt *b*_562_^N80C^)_n_ and apoHTHP under equimolar conditions with respect to the amount of heme-binding site, (**B**) fractions collected in the shaded region of A, (**C**) a mixture of apoHTHP and three equivalents of (**1**-Cyt *b*_562_^N80C^)_n_ with respect to the amount of heme-binding site, and (**D**) fractions collected in the shaded region of C. These traces were monitored by absorbance at 402 nm. (**E**) SEC traces of 10 μM of (**1**-Cyt *b*_562_^N80C^)_n_ assembly in 0.1 M potassium phosphate buffer at pH 7. This chromatogram was monitored by absorbance at 418 nm. (**F**) SEC traces of the authentic samples monitored by the absorbance at 280 nm: ferritin (474 kDa), albumin (66.5 kDa), and chymotrypsin (25.6 kDa). Building block proteins, apoHTHP (36 kDa) and Cyt *b*_562_ (12 kDa), are also shown and monitored by absorbance at 280 nm and 418 nm, respectively.

The size of the assembly in the crude mixture generated by mixing equimolar (**1**-Cyt *b*_562_^N80C^)_n_ and apoHTHP with respect to the amount of heme binding site at 45 °C was evaluated by size exclusion chromatography (SEC) analysis. The elution volume of a major SEC peak of this assembly is 14.4 mL (Figure 5A), whereas the elution volumes of Cyt *b*_562_ and apoHTHP are 17.2 mL [29] and 16.0 mL, respectively [21]. This result indicates the existence of a structure which is larger relative to Cyt *b*_562_ and apoHTHP. From our previous study [18], the size range of (**1**-Cyt *b*_562_^N80C^)_n_ is mainly composed of oligomers of 5-mer to 50-mer. This is because its initial elution volume in the SEC trace in Figure 5E corresponds to >474 kDa. Compared to (**1**-Cyt *b*_562_^N80C^)_n_, the assembly is smaller because of dissociation of the linear assembly upon addition of apoHTHP. Fractionation of the shaded region indicated in Figure 5A was performed to collect major components of the target structures. Analysis of the fractionated components shows the presence of the maintained assembly and absence of small proteins such as Cyt *b*_562_ and apoHTHP elution over 16 mL, while tailing of the peak is observed. The smaller components in the tail of the peak represent insignificant populations, which may be derived from the dissociation of the supramolecular assembly by dilution, during the fractionation, due to instability (Figure 5B). The fractionated component, 1/1-(**1**-Cyt *b*_562_^N80C^)_n_-apoHTHP assembly, where n in n/1 denotes the equivalence of **1**-Cyt *b*_562_^N80C^ towards the heme-binding sites of apo-HTHP in the preparation stage, was further evaluated as described below.

Mixing apoHTHP and three equivalents of (**1**-Cyt *b_562_*^N80C^)_n_ with respect to the amount of heme-binding site at 45 °C also forms large assemblies which were confirmed by SEC (Figure 5C). In comparison to the equimolar mixture, the SEC trace has an earlier elution volume of 13.9 mL (Figure 5D), indicating a larger structure with a molecular mass clearly greater than 66.5 kDa with respect to the authentic samples (Figure 5F), namely ferritin (474 kDa), albumin (66.5 kDa), and chymotrypsin (25.6 kDa). The assemblies were also fractionated in the shaded region of Figure 5C and SEC analysis indicates that the large assemblies are maintained after the fractionation. The fractionated component, 3/1-(**1**-Cyt *b*_562_^N80C^)_n_-apoHTHP assembly, was also evaluated in a manner similar to the evaluation of the 1/1-(**1**-Cyt *b*_562_^N80C^)_n_-apoHTHP assembly.

#### 2.2.3. UV-Vis Spectra of Fractionated Assemblies

**Figure 6:**
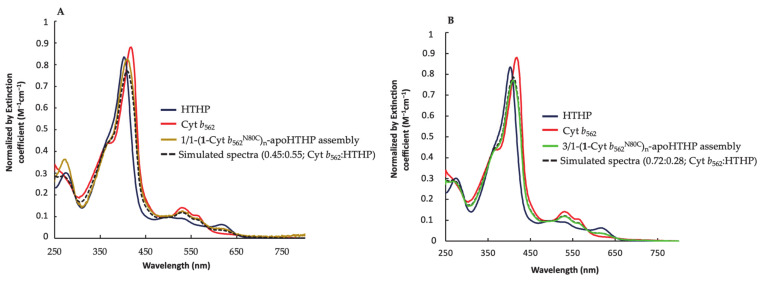
(**A**) UV-Vis spectra of 1/1-(**1**-Cyt *b*_562_^N80C^)_n_-apoHTHP assembly in brown, wild-type HTHP in navy blue and (**1**-Cyt *b*_562_^N80C^)_n_ in red. The simulated spectrum is the dotted black line. (**B**) UV-Vis spectra of 3/1-(**1**-Cyt *b*_562_^N80C^)_n_-apoHTHP assembly in green, wild-type HTHP in navy blue and (**1**-Cyt *b*_562_^N80C^)_n_ in red. The simulated spectrum is the dotted black line.

A representative UV-Vis spectrum of the 1/1-(**1**-Cyt *b*_562_^N80C^)_n_-apoHTHP assembly is shown in Figure 6A. The UV-Vis spectrum provides the ratio of heme moieties incorporated into heme-binding sites of HTHP and **1**-Cyt *b*_562_^N80C^. The spectrum was analyzed by a simulation using UV-Vis spectra of wild-type HTHP and (**1**-Cyt *b*_562_^N80C^)_n_. The spectra in Figure 6A are normalized by extinction coefficients and a simulated spectrum was generated by maintaining the sum of the factor multiplication of two spectra of wild-type HTHP and (**1**-Cyt *b*_562_^N80C^)_n_ to 1. The best-fitted simulation in the region attributed to heme absorption was obtained with a ratio of 0.45:0.55 for **1**-Cyt *b*_562_^N80C^:HTHP (Figure 6A). Similarly, 3/1-(**1**-Cyt *b*_562_^N80C^)_n_-apoHTHP assembly results in a UV-Vis spectrum corresponding to a simulation of 0.72: 0.28 for **1**-Cyt *b*_562_^N80C^:HTHP (Figure 6B).

#### 2.2.4. SDS-PAGE of Fractionated Assemblies

**Figure 7:**
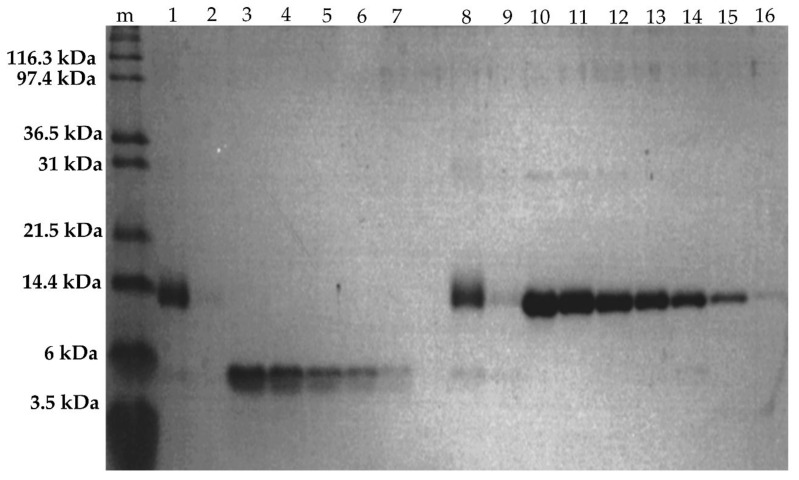
SDS-PAGE for protein concentration analysis. Lanes 1 and 8: 3/1-(**1**-Cyt *b*_562_^N80C^)_n_-apoHTHP assembly. Lanes 2 and 9: 3/1-(**1**-Cyt *b*_562_^N80C^)_n_-apoHTHP assembly. Lanes 3–7: 10 μM, 8 μM, 6 μM, 4 μM, and 2 μM of HTHP in lanes 3, 4, 5, 6, 7, respectively. Lanes 10–16: 30 μM, 25 μM, 20 μM, 15 μM, 10 μM, 5 μM, and 2 μM of **1**-Cyt *b*_562_^N80C^ in lanes 10, 11, 12, 13, 14, 15, and 16, respectively.

The analysis of UV-Vis spectra only provides information regarding the presence of heme moieties. To estimate the components in the fractionated assembly, the ratio of the protein matrices of HTHP and **1**-Cyt *b*_562_^N80C^ are required. SDS-PAGE enables evaluation of the amount of each of the denatured monomeric proteins of HTHP and **1**-Cyt *b*_562_^N80C^ (Figure 7). SDS PAGE results of the fractionated assemblies revealed the presence of both proteins, **1**-Cyt *b*_562_^N80C^ and HTHP, according to bands of their molecular weights, of about 12 kDa and about 6 kDa, respectively. The density of each band was quantified by image analysis. The density depends on the concentration of monomeric proteins’ interaction with the staining molecule, Coomassie Brilliant Blue (CBB), employed in the analysis. The strength of the interaction between CBB and protein is specific to each protein. Thus, calibration curves for apoHTHP and **1**-Cyt *b*_562_^N80C^ were prepared to determine the concentration of monomeric proteins in the fractionated samples. Here, the fractionated samples and standards for the calibration curve on the same electrophoresis gel were analyzed to minimize the deviation. Protein samples of known various concentrations with the same volumes were evaluated by SDS-PAGE and the band densities were analyzed by image analysis. In the range from 2 to 10 µM of the HTHP monomer, a linear relationship exists in the plots of the peak area of intensity derived from band density against the protein concentration (Figure S1, Table S1). Similarly, SDS-PAGE of **1**-Cyt *b*_562_^N80C^ of increasing concentrations from 2 μM to 30 μM shows a linear relationship of the peak area of intensity derived from band density against the protein (Figure S2, Table S2). Although the area intensities include some deviations around 10–20%, acceptable correlations were obtained in both calibration curves. Thus, these experiments will show the moderate accuracy to determine the protein concentrations. An analysis of the sample mixtures to obtain the ratio of **1**-Cyt *b*_562_^N80C^ and apoHTHP present in 1/1-(**1**-Cyt *b*_562_^N80C^)_n_-apoHTHP assembly was carried out using the peak area of intensity derived from band densities and a calibration curve for each protein. The concentrations of **1**-Cyt *b*_562_^N80C^ and apoHTHP in the fractionated components were determined to be 4.7 ± 0.19 and 2.6 ± 0.44 µM as monomers, respectively (Table 1). Since HTHP always forms a hexamer, an average of 10 to 11 units of **1**-Cyt *b*_562_^N80C^ are conjugated to each hexamer of apoHTHP. In a similar manner, the concentrations of **1**-Cyt *b*_562_^N80C^ and apoHTHP in 3/1-(**1**-Cyt *b*_562_^N80C^)_n_-apoHTHP assembly were determined to be 8.6 ± 1.2 and 3.2 ± 0.51 µM as monomers, respectively (Table 1). Thus, an average of 16 units of **1**-Cyt *b*_562_^N80C^ are assembled with each hexamer of apoHTHP. 

#### 2.2.5. Estimation of Apparent Structures of (1-Cyt b_562_^N80C^)_n_-apoHTHP Assemblies

**Figure 8:**
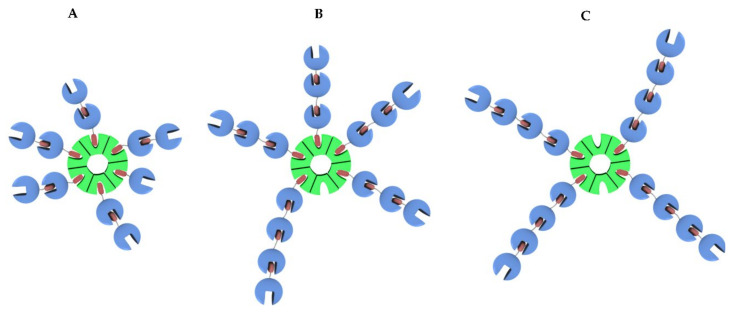
Schematic representation of one of the examples of structures for 1/1-(**1**-Cyt *b*_562_^N80C^)_n_-apoHTHP assembly (**A**) and two examples of the structures for 3/1-(**1**-Cyt *b*_562_^N80C^)_n_-apoHTHP assembly (**B**,**C**).

Estimations of the ratios of the monomer units and location of the bound heme moieties provide the apparent structure of the fractionated components. In the 1/1-(**1**-Cyt *b*_562_^N80C^)_n_-apoHTHP assembly, the HTHP hexamer has a fully occupied heme-binding site with five **1**-Cyt *b*_562_^N80C^ dimers and one **1**-Cyt *b*_562_^N80C^ monomer. This arrangement is in agreement with the experimental results of 11:6 monomer units ratio and a 0.45:0.55 location ratio of bound heme moieties for **1**-Cyt *b*_562_^N80C^:apoHTHP. An example of the estimated structures is described in Figure 8A. In the 3/1-(**1**-Cyt *b*_562_^N80C^)_n_-apoHTHP assembly, experimental results of a 16:6 monomer units ratio and a 0.72:0.28 location ratio of bound heme moieties for **1**-Cyt *b*_562_^N80C^:HTHP suggest the formation of an assembly of HTHP hexamer having one vacant heme-binding site with one **1**-Cyt *b*_562_^N80C^ tetramer and four **1**-Cyt *b*_562_^N80C^ trimers (an example is shown in Figure 8B). Another possibility is an assembly of HTHP hexamer with two vacant heme-binding sites and four **1**-Cyt *b*_562_^N80C^ tetramers (an example is shown in Figure 8C). Although the estimated structures are apparent, the presence of vacant heme-binding sites of HTHP in the presence of excess **1**-Cyt *b*_562_^N80C^ would be caused by steric hindrance with adjacent **1**-Cyt *b*_562_^N80C^ units. Both proteins are cylindrical in shape with different sizes. HTHP has a diameter of 5–6 nm and a height of 5 nm, while Cyt *b*_562_ has a diameter of 2.5 nm and a height of 5 nm. Thus, the longer side of **1**-Cyt *b*_562_^N80C^ bound to apoHTHP appears to induce steric hindrance, which prevents the interaction between the adjacent heme binding sites of HTHP and the heme moiety of **1**-Cyt *b*_562_^N80C^ in the assembly system. Although further purification and analysis by high-resolution TEM or AFM is required to determine the detailed structures and the populations, the examples of the apparent assemblies, as shown in Figure 8, were qualitatively proposed by the analysis of SDS-PAGE and UV-vis spectra. 

#### 2.2.6. Evaluation of Hydrodynamic Diameter by Dynamic Light Scattering Analysis 

**Figure 9:**
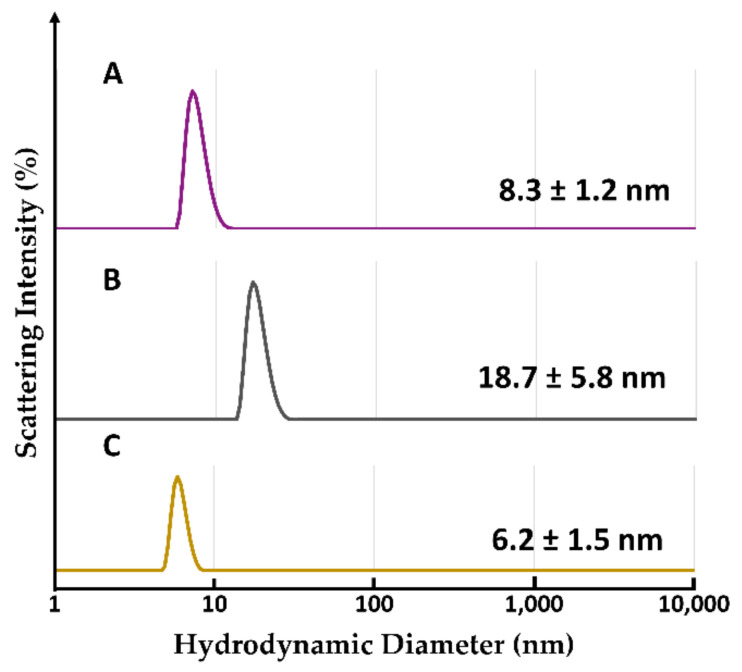
Hydrodynamic diameter distributions for (**A**) 1/1-(**1**-Cyt *b*_562_^N80C^)_n_-apoHTHP assembly, (**B**) 3/1-(**1**-Cyt *b*_562_^N80C^)_n_-apoHTHP assembly and (**C**) apoHTHP.

The hydrodynamic diameters of the fractionated components were measured by dynamic light scattering (DLS). Each fractionated solution was concentrated to 20 µM based on heme concentration to provide sufficient light scattering intensity. The distribution of hydrodynamic diameters for the fractionated assemblies (Figure 9A, Figure 9B) and apoHTHP as shown in Figure 9C. Hydrodynamic diameters of the fractionated components are obviously larger than the apoHTHP hydrodynamic diameter of 6 nm and reported Cyt *b*_562_ diameter of ca. 2.5 nm [26]. The estimated structure of 1/1-(**1**-Cyt *b*_562_^N80C^)_n_-apoHTHP assembly roughly suggests a maximum hydrodynamic diameter of about 16 nm (one HTHP: 6 nm + two **1**-Cyt *b*_562_^N80C^ dimers: 2 × 2 × 2.5 nm) when HTHP and **1**-Cyt *b*_562_^N80C^ have a flat arrangement providing a maximum size. However, the experimental hydrodynamic diameter of this fractionated component is 8.3 nm, indicating that the structure is not flat and is likely flexible in solution. Similarly, the maximum diameter of 3/1-(**1**-Cyt *b*_562_^N80C^)_n_-apoHTHP assembly is estimated to be about 26 nm (one HTHP: 6 nm + two **1**-Cyt *b*_562_^N80C^ tetramers: 2 × 4 × 2.5 nm or one HTHP: 6 nm + one **1**-Cyt *b*_562_^N80C^ tetramers: 4 × 2.5 nm + one 1-Cyt *b*_562_^N80C^ trimer: 3 × 2.5 nm) in the flat arrangement and this value is greater than the experimentally-determined hydrodynamic diameter of 18.7 nm. In both assemblies, the experimentally-determined hydrodynamic diameters are reasonable when flexible arrangements of (**1**-Cyt *b*_562_^N80C^)_n_ moieties are hypothesized.

## 3. Materials and Methods 

### 3.1. Instruments and Materials

UV-Vis absorption spectra were measured using a Shimadzu UV-2700 or a Shimadzu BioSpec-nano spectrophotometer. The pH values were recorded with a Horiba F-52 pH meter. Size exclusion chromatographic (SEC) analyses were performed using a Superdex 200 Increase 10/300 GL (GE Healthcare, Chicago, IL, USA) column with ÄKTA pure 25 (GE Healthcare, Chicago, IL, USA) at 4 °C. Dynamic light scattering (DLS) measurements were carried out by a Zetasizer μV (Malvern Instruments, Malvern, UK) with an 830 nm laser at 25 °C. Ultrapure water (milli-Q) was prepared by a Merck Millipore Integral 3 apparatus. Cyt *b*_562_, (**1**-Cyt *b*_562_^N80C^)_n_ and apoHTHP are prepared according to our reported methods [18]. All other reagents were commercially available and used as received or otherwise specified.

### 3.2. Preparation of Unmodified Cyt b_562_ with apoHTHP

One equivalent of Cyt *b*_562_ unmodified (0.5 mL,10 μM) was added to a solution of apoHTHP (0.5 mL, 10 μM as a monomer) in 100 mM potassium phosphate buffer pH 7.0 and mixed at 45 °C for 4 h. The protein solution was cooled to room temperature and characterized by UV-Vis spectroscopy. 

### 3.3. Preparation of Assembly by apoHTHP and Equimolar (1-Cyt b_562_^N80C^)_n_

One equivalent of (**1**-Cyt b_562_^N80C^)_n_ (0.5 mL, 10 μM as a monomer) was added to a solution of apoHTHP (0.5 mL, 10 μM as a monomer) in 100 mM potassium phosphate buffer at pH 8.0 and mixed at 45 °C for 4 h. The protein solution was cooled to room temperature and characterized by UV-Vis spectroscopy and SEC.

### 3.4. Preparation of Assembly Produced by apoHTHP and the Three Equivalent (1-Cyt b_562_^N80C^)_n_

Three equivalent of (**1**-Cyt b_562_^N80C^)_n_ (0.5 mL, 30 μM as a monomer) was added to a solution of apoHTHP (0.5 mL, 10 μM as a monomer) in 100 mM potassium phosphate buffer pH 8.0 and mixed at 45 °C for 4 h. The protein solution was cooled to room temperature and characterized by UV-Vis spectroscopy and SEC.

### 3.5. SEC Analyses, Sample Preparations and SEC Fractionation

For SEC analysis, 100 mM potassium phosphate buffer at pH 7.0 was used as an eluent. The analysis was carried out at 4 °C at a flow rate of 0.5 mL/min with absorbance being monitored at 280 nm, 418 nm, and 402 nm for detection. The Superdex 200 Increase 10/300 GL column (GE Healthcare, Chicago, IL, USA) was calibrated using: ovalbumin (45 kDa), albumin (66.5 kDa), and chymotripsinogen (25.6 kDa). Sample solutions were filtered through a Millex-GV Syringe Driven Filter Unit 0.22 μm diameter and 100 μL of filtered sample solution is used for SEC analyses. The same column, eluent, flow rate, temperature and absorbance were used for fractionation settings. The fixed fractionation volume was set to 0.5 mL and eluted fractions were collected in a 96-well plate at 4 °C. 

### 3.6. SDS-PAGE

SDS-PAGE was conducted using a 12% separating gel and a 4% stacking gel. The collected fractions were concentrated using an Amicon Ultra Centrifugal Filter (5 mL tube and 10 kDa cut-off) and 5 μL was mixed with an equal volume of loading buffer containing 50% 10 mM Tris-HCl buffer, 10% 2-mercaptoethanol, 4% SDS, 10% sucrose, and 0.05% bromophenol blue with addition of milli-Q up to 10 μL. The electrophoresis was run at 150 V, 120 mA, 18 W at 60 ± 10 min. 

### 3.7. Protein Quantification by Image Analysis of SDS-PAGE

Image analyses of SDS-PAGE were carried out using Image J Software. From the gels option in the analyze tools section, the first lane was selected corresponding to the lowest concentration of protein of interest (in this case **1**-Cyt *b*_562_^N80C^, HTHP or samples). After the first lane was chosen, a rectangle with the same shape and size as the first lane was applied to the other lanes to provide exactly the same size, space, noise and defined area for each band analysis. For each lane, the plot of intensity of band density against migration distance was generated. The area of the peak in each plot with linear baseline correction was determined. The peak areas were used to obtain calibration curves to evaluate the concentrations of HTHP and **1**-Cyt *b*_562_^N80C^. 

### 3.8. Hydrodynamic Diameter by DLS Measurement

For DLS measurements, an aqueous solution of sample was concentrated using an Amicon Ultra Centrifugal Filter (5 mL tube and 10 kDa cut-off). To analyze the protein solution, a 12 μL quartz cuvette was used. The sample solution was filtered through a 0.22 μm syringe driven filter unit before pipetting into a cuvette. The data were obtained by the number-based particle size distribution mode. 

## 4. Conclusions

Cyt *b*_562_^N80C^-based linear assembly, (**1**-Cyt *b*_562_^N80C^)_n_, was assembled with hexameric heme binding protein, apoHTHP, to form supramolecular star-shaped structures via the heme–heme pocket interactions. Because of the slow equilibrium for transfer of heme moieties, heating at 45 °C is required for efficient formation of the assembly. The mixing ratio of the protein units dominantly controls the assembled structures, which were experimentally estimated as apparent structures based on UV-Vis spectroscopy and SDS-PAGE analyses. In one example of the assembled structures, four **1**-Cyt *b*_562_^N80C^ tetramers are bound to one apoHTHP hexamer. The clearly distinguished UV-Vis spectra of Cyt *b*_562_ and HTHP enable these estimations, indicating that hemoproteins are useful building blocks to provide information regarding the assembly compositions in this system, which includes multiple components. The present system successfully provides a star-shaped hemoprotein assembly using oligomeric hemoprotein as a core unit, in contrast to our previous reports of a heme trimer and Cyt *b*_562_-based linear assembly, which generates a large network structure on the substrate [15]. This difference is clearly derived from the absence of the heme–heme interaction between redundant exposed heme moieties in this work: termination of a linear assembly by a vacant heme pocket of **1**-Cyt *b*_562_^N80C^ is favorable for generation of the star-shaped structure. The obtained assembly is expected to provide a useful scaffold with potential for development of new classes of nanobiomaterials in applications such as light harvesting antennas.

## Figures and Tables

**Table 1:** Concentration of protein components in the fractionated assemblies.

Assembly	Components	Concentration as a Monomer (μM)
1/1-(**1**-Cyt *b*_562_^N80C^)_n_-apoHTHP assembly	**1**-Cyt *b*_562_^N80C^	4.7 ± 0.19
	apoHTHP	2.6 ± 0.44
3/1-(**1**-Cyt *b*_562_^N80C^)_n_-apoHTHP assembly	**1**-Cyt *b*_562_^N80C^	8.3 ± 1.2
	apoHTHP	3.2 ± 0.51

## Supplementary Materials

Supplementary Materials can be found at https://www.mdpi.com/1422-0067/22/3/1012/s1. 1: Calculation to determine the concentration of protein units by image analysis of SDS-PAGE, Figure S1: Image analysis of SDS-PAGE for HTHP and relationship between band density and HTHP concentration, Figure S2: Image analysis of SDS-PAGE for **1**-Cyt *b*_562_^N80C^ and relationship between band density and **1**-Cyt *b*_562_^N80C^ concentration, Figure S3: Image analysis of SDS-PAGE for assemblies, Table S1: Peak areas of SDS-PAGE bands for various concentration of HTHP, Table S2: Peak areas of SDS-PAGE bands for various concentration of **1**-Cyt *b*_562_^N80C^, Table S3: Peak areas of SDS-PAGE bands for assemblies

## Abbreviations

SDS-PAGE	Sodium dodecyl sulfate-polyacrylamide gel electrophoresis
PDB	Protein Data Bank
TEM	Transmission electron microscopy
AFM	Atomic force microscopy
