# Supplementary material for: A Supramolecular Assembly of Hemoproteins Formed in a Star-Shaped Structure via Heme–Heme Pocket Interactions
Source: Int J Mol Sci. 2021 Jan 20;22(3):1012. doi: 10.3390/ijms22031012 (PMC7864044; doi:10.3390/ijms22031012)
Supplement: Supplementary file 1 [file ijms-22-01012-s001.pdf]

A Supramolecular Assembly of Hemoproteins Formed in a Star-Shaped Structure via Heme-Heme Pocket Interactions

Julian Wong Soon, Koji Oohora,* Shota Hirayama, and Takashi Hayashi*

ELECTRONIC SUPPLEMENTARY INFORMATION

Table of contents:

1. Figure S1. Image analysis of SDS-PAGE for HTHP and relationship between band density and HTHP concentration.
2. Figure S2. Image analysis of SDS-PAGE for **1**-Cyt b_{562}^{N80C} and relationship between band density and **1**-Cyt b_{562}^{N80C} concentration.
3. Figure S3. Image analysis of SDS-PAGE for assemblies.
4. Table S1. Peak areas of SDS-PAGE bands for various concentration of HTHP.
5. Table S2. Peak areas of SDS-PAGE bands for various concentration of **1**-Cyt b_{562}^{N80C}.
6. Table S3. Peak areas of SDS-PAGE bands for assemblies.

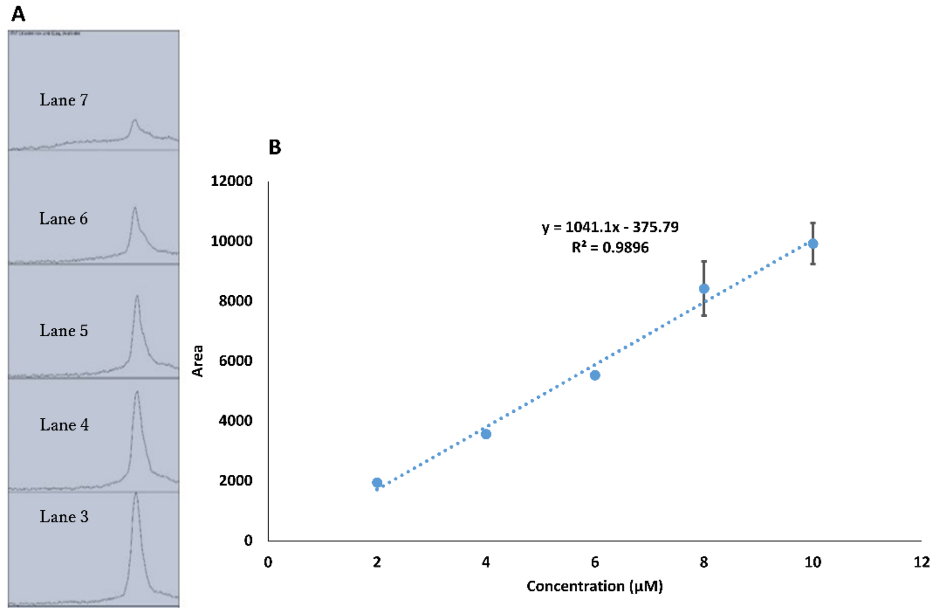

Figure S1: (**A**) Plots of peak area of the intensity derived from band density against the HTHP concentration as a monomer. (**B**) Plots of intensity derived from band density against migration distance by image analysis for lanes 3-7 of SDS-PAGE in Figure 7. Broken line shows corresponding least square fitting.

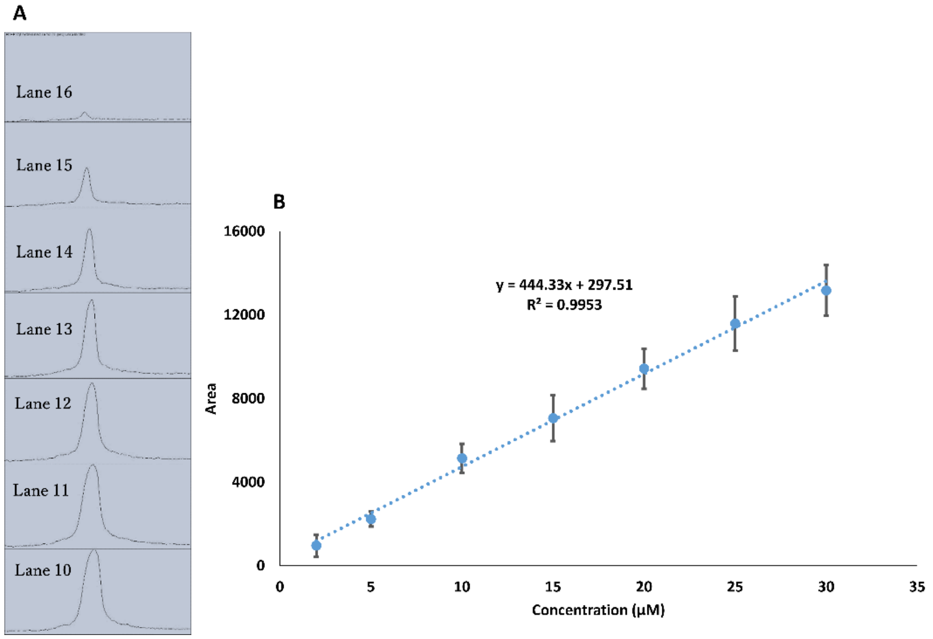

Figure S2. (**A**) Plots of peak area of the intensity derived from band density against the concentration of **1**-Cyt b_{562}^{N80C}. (**B**) Plots of intensity derived from band density against migration distance by image analysis for lanes 10-16 of SDS-PAGE in Figure 7. Broken line shows corresponding least square fitting.

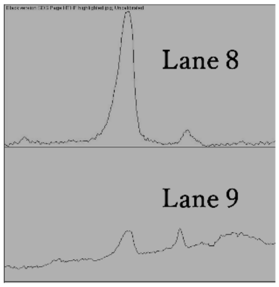

Figure S3. Plots of intensity derived from band density against migration distance by image analysis for lanes 8 and 9 of SDS-PAGE in Figure 7.

Table S1: Peak areas of SDS-PAGE bands for various concentration of HTHP.

Lane	Concentration as a monomer (μM)	Peak area[1]
3	10	9919 ± 674
4	8	8417 ± 897
5	6	5525 ± 103
6	4	3556 ± 99
7	2	1937 ± 65

[1]The error for each peak area was obtained from the standard deviation of three separate SDS PAGE experiments.

Table S2. Peak areas of SDS-PAGE bands for various concentration of **1**-Cyt b_{562}^{N80C}.

Lane	Concentration (μM)	Peak area[1]
10	30	13176 ± 1201
11	25	11594 ± 1292
12	20	9436 ± 960
13	15	7066 ± 1095
14	10	5142 ± 698
15	5	2238 ± 363
16	2	970 ± 105

[1]The error for each peak area was obtained from the standard deviation of three separate SDS PAGE experiments.

Table S3. Peak areas of SDS-PAGE bands for assemblies.

Lane	Assembly	Components	Peak areas[1]	Concentration as a monomer (μM)[2]
9	1/1-(**1**-Cyt b_{562}^{N80C})$_n$-apoHTHP assembly	**1**-Cyt b_{562}^{N80C}	2392 ± 214	4.7 ± 0.19
		apoHTHP	2312 ± 83	2.6 ± 0.44
8	3/1-(**1**-Cyt b_{562}^{N80C})$_n$-apoHTHP assembly	**1**-Cyt b_{562}^{N80C}	4125 ± 825	8.3 ± 1.2
		apoHTHP	3047 ± 153	3.2 ± 0.51

[1]The error for each peak area was obtained from the standard deviation of three separate SDS PAGE experiments. [2]Concentrations were determined from the peak areas and calibration curve for each protein.
